# Hydration and Strength Development of Cementitious Materials Prepared with Phosphorous-Bearing Clinkers

**DOI:** 10.3390/ma14030508

**Published:** 2021-01-21

**Authors:** Lilan Xie, Min Deng, Jinhui Tang, Kaiwei Liu

**Affiliations:** 1 College of Materials Science and Engineering, Nanjing Tech University, Nanjing 211816, China; 2 College of Materials and Construction Engineering, Guizhou Normal College, Guiyang 550018, China; 3 School of Materials Science and Engineering, Southeast University, Nanjing 211189, China; t_jinhui@163.com; 4 Anhui Province Engineering Laboratory of Advanced Building Materials, Anhui Jianzhu University, Hefei 230601, China; liukaiwei1985@gmail.com

**Keywords:** phosphorous-containing cement, α′-C_2_S-xC_3_P, mechanical property, hydration mechanism

## Abstract

To rationally use low-grade phosphorous limestone as the raw materials for cement production, the influence of phosphorous introduced by fluorapatite during the clinker calcination process on the mechanical properties of cementitious materials is investigated. Hydration kinetics, phase evolutions, and microstructure of cement pastes have been studied by using calorimetry, X-ray diffraction (XRD), and scanning electron microscopy (SEM). The results indicate that the mechanical properties of cementitious materials can be slightly improved due to the mineralization effect of the small amount of phosphorous in the clinker and significantly decreased with an increase of phosphorous. High content of phosphorous will reduce the content of C_3_S and make the formation of α′-C_2_S-xC_3_P(x: 0–0.05), whose hydration reactivity is rather lower, such that on the one hand less-hydrated products, such as calcium silicate hydrate (C-S-H) gel, can be obtained, and on the other hand, the hydration reaction will be slowed by severely prolonging the induction period. Interestingly, small particles can be observed on the surface of hydration products, but no new phase can be detected by XRD. When the content of P_2_O_5_ is 2.0%, the cement can meet the requirements of P·II 42.5 cement in China. Hopefully, this can provide significant guidance for the use of cement prepared by fluorapatite as raw material.

## 1. Introduction

Phosphorus has a significant effect on the hydration performance of cement, such as the hydration rate of cement clinker, and the strength of cement-based materials [1,2,3,4,5]. Phosphorus in cement usually comes from two sources; one is phosphorus introduced in the raw materials for cement production or alternative fuels [6,7,8], and the other is the partial replacement of cement by solid waste containing phosphorus (phosphorus slag, phosphor-gypsum, phosphorus tailings, etc.) [9,10,11].

The phosphorus contained in cement clinker is usually called internal phosphorus, while other phosphorus introduced in cement is called external phosphorus. Many studies have been conducted on the influence of externally introduced phosphorus on cement hydration performance [12,13,14,15,16]. Lieber [1] used dipolyphosphate, tripolyphosphate, tetrapolyphosphate, and polyphosphate to study the effects of different soluble phosphates on cement hydration. According to the properties and content of the decomposed phosphate in the mixed solution, it is considered that phosphate ions adsorb particles on the surface of the clinker and the formation of calcium phosphate complexes probably prevents the normal process of hydration. Chen et al. [3] mixed chemical reagent P_2_O_5_ with Portland cement to study the influence of P_2_O_5_ on the hydration mechanism of Portland cement. The results show that when the P_2_O_5_ content is up to 3.5%, the total heat of hydration of Portland cement is reduced by 32.6%, and the initial and final settings of Portland cement are delayed by 1.10 h and 12.54 h, respectively. The reason for this is that new hydration products form during the hydration of C_3_A, which increases hydration resistance and apparent activation energy of cement during the acceleration period, and delays the hydration process of C_3_S and β-C_2_S.

The mechanism of retardation of poorly soluble phosphate and soluble phosphate on cement is different. Cheng et al. [17] believe that the insoluble phosphate is gradually converted into hydroxyapatite in alkaline solution and adsorbed on the surface of the hydration product, resulting in an increase in the compactness of the hydration product film. Boughanmi [10] conducted research on phosphorus-containing industrial clinker in Tunisia, and the results showed that when the content of P_2_O_5_ in the clinker is 0.5–1.1%, it has no significant effect on the hydration activity and strength development of cement. “International Cement Process Information Collection” [18] mentioned that the allowable content of P_2_O_5_ in the clinker is about 2.5% if raw materials containing phosphorus. Sometimes, the content of P_2_O_5_ is very low, even as low as 0.5%, but it still causes a significant decrease in cement strength, especially the early strength of cement.

Therefore, it is interesting to investigate the hydration and strength development of cementitious materials prepared with phosphorous-bearing clinker. In fact, lots of work regarding the influence of external addition of phosphors by chemical agent on the property of cement has been done in the lab, which is not closely related with real-world situations. At present, the hydration and mechanical properties of Portland cement clinker prepared with limestone containing fluorapatite as raw material are rarely reported. In this paper, phosphorus-containing limestone, from a cement plant in Angola, was used to prepare cement clinker, and furthermore to study the effect of phosphorus on the hydration activity of cement clinker, and the influence of phosphorous in cement clinker on the phase evolution and morphology. Moreover, the degree of hydration of the cement was studied through calorimetry, TG-DSC, and mechanical performance testing. The results can be used as the guidance for cement production using low-grade phosphorous-bearing limestone.

## 2. Experimental

### 2.1. Materials

#### 2.1.1. Cements

Clinkers with different phosphorus contents were experimentally prepared with raw reals obtained by mixing phosphorus-containing limestone, normal limestone, clay, iron ore powder, and fly ash. The phosphorus-containing limestone was derived from Angola (Cimangola, Luanda, Angola). KH (lime saturation factor, SM (silica modulus), and IM (iron modulus) for clinkers were 0.96, 2.10, and 1.50. The temperature for calcining clinkers was 1450 °C. The content of P2O5 in the clinkers was 0.0%, 0.5%, 1.0%, 1.5%, 2.0%, and 2.5% by adjusting the mass ratio of phosphorus-containing limestone and limestone. The chemical compositions of clinkers obtained by XRF (X-ray Fluorescence) are shown in Table 1. The content of C_3_S and C_2_S (including β-C_2_S and α′-C_2_S-xC_3_P) was measured by Retiveld refinements of powder XRD. Mixtures of 95% clinkers and 5% natural gypsum were ground in a lab mill to obtain cements. The particles of the obtained cements all passed through 80 μm sieve. 

#### 2.1.2. Sand

**Figure 1:**
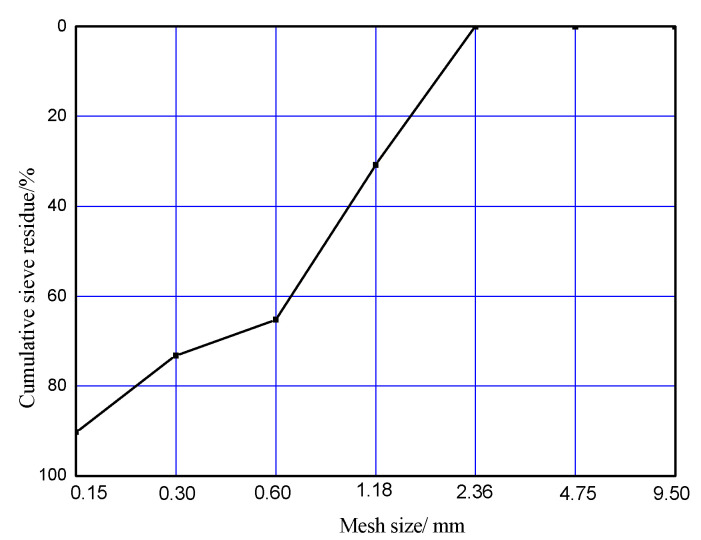
The gradation diagram of sand.

Standard sand from Pingtan, Fujian province, China was used. The purity of the sand used in this paper was higher than 98%. The particle size varied from 0.08 mm–2.00 mm, which is shown in Figure 1.

### 2.2. Test Methods

#### 2.2.1. Hydration Heat

The TAM AIR Thermstate 90 thermal activity microcalorimeter of American TA Company (New Castle, DE, USA) was used for calorimetric analysis. The water–binder ratio of the test sample was 0.5 and the mass of cement was about 1.00 g. The temperature was kept at 20 °C.

#### 2.2.2. Phase Compositions of Cement Pastes

The cement pastes at certain curing ages were crushed and soaked in ethyl alcohol for 2 days to stop hydration. They were then ground to pass through an 80 µm sieve and vacuum-dried at 70 °C. The hydration products of cement pastes were determined by SmartLab rotary target X-ray diffractometer (Rigaku, Tokyo, Japan) equipped with copper, as were the target materials from Rigaku Company, Japan. The scanning rate was 10°/min. Working voltage and current were 40 kV and 30 mA. A STA449F3 thermal analyzer (NETCSZH, Selb, Germany) was used to analyze the phases of cement pastes with N_2_ protective atmosphere. The reference substance was α-Al_2_O_3_. The samples were heated from room temperature to 900 °C at a rate of 10 °C/min.

#### 2.2.3. Microstructures

A S-4800 scanning electron microscope produced by HITACHI (Tokyo, Japan) was used to analyze the microstructure of cement pastes at different ages.

#### 2.2.4. Strength of Cement Pastes and Mortars

Cement pastes of 20 mm × 20 mm × 20 mm were cast with 0.28 W/C. They were then cured in a moist container at 20 °C ± 2 °C for 24 h. They were de-molded and immersed in 20 °C water. Compressive strengths of the cement pastes cured in water for 2 and 27 days were tested. 

Mortars of 40 mm × 40 mm × 160 mm were cast with cements, sand, and water. The ratio of cement to sand was 1:3. W/C was 0.50. The mortars were then cured in a moist container at 20 °C ± 2 °C for 24 h. They were de-molded and immersed in 20 °C water. The compressive and flexural strengths of mortars cure for 3 and 28 days were tested according to GB/T 17671-1999 “Test method for strength of mortars”. A detailed proportion of both cement pastes and mortars is listed in Table 2.

## 3. Results and Discussion

### 3.1. Heat of Hydration

**Figure 2:**
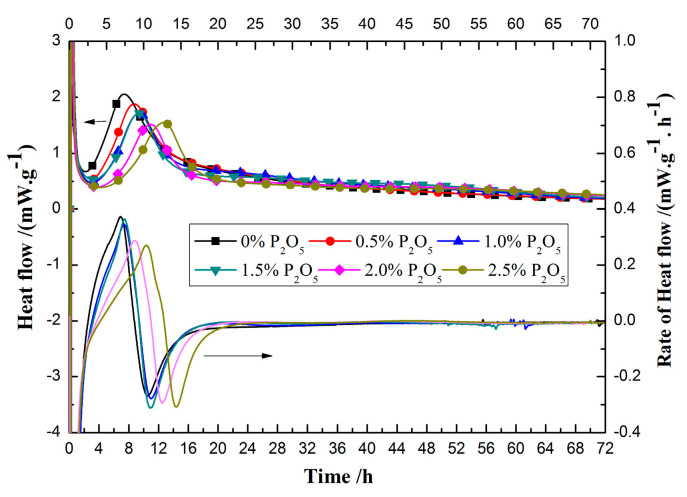
Influences phosphorus on the hydration heat of cement.

To study the effect of P_2_O_5_ content on the hydration process of cement, calorimetric analysis was carried out on the prepared cement samples, as shown in Figure 2. The characteristic values of the hydration heat curve of phosphorous-containing cement are listed in Table 3.

From Figure 2, compared with plain sample, the induction period for those phosphorous-containing cement will be prolonged. In addition, the higher the P_2_O_5_, the longer the induction period. Moreover, the maximum hydration heat flow decreased with the increase of the content of P_2_O_5_.

According to characteristic points in the hydration heat curve of cement, the hydration heat curve is divided into the following stages [19]: the end of the induction period, which means the beginning of the acceleration period, and the end of the acceleration period, when the heat release rate reaches maximum value. Based on the test results of hydration heat in Figure 2, the characteristic parameters of hydration heat curves of cement with different P_2_O_5_ contents are listed in Table 3.

The induction period was prolonged separately by 0.51 h, 0.86 h, 1.15 h, 1.55 h, and 3.28 h when the content of P_2_O_5_ in the clinker increased from 0.5% to 2.5%. With an increase of P_2_O_5_ content in the clinker, the minimal hydration heat flow decreases gradually. When the P_2_O_5_ content in the clinker is up to 2.5%, the hydration heat flow decreases to 0.40 mW·g^−1^ in induction, which means the existence of phosphorous will slow down the reaction rate of cement clinker. At the same time, the maximum hydration heat flow also decreases with the increase of P_2_O_5_ content in the clinker. Total hydration heat will first increase with the increase of the P_2_O_5_ content. However, total hydration heat will decrease if the content of P_2_O_5_ is higher than 2.0%, which may be closely related to the total content of silicate phase in cement.

### 3.2. Hydration Products in Cement Pastes

**Figure 3:**
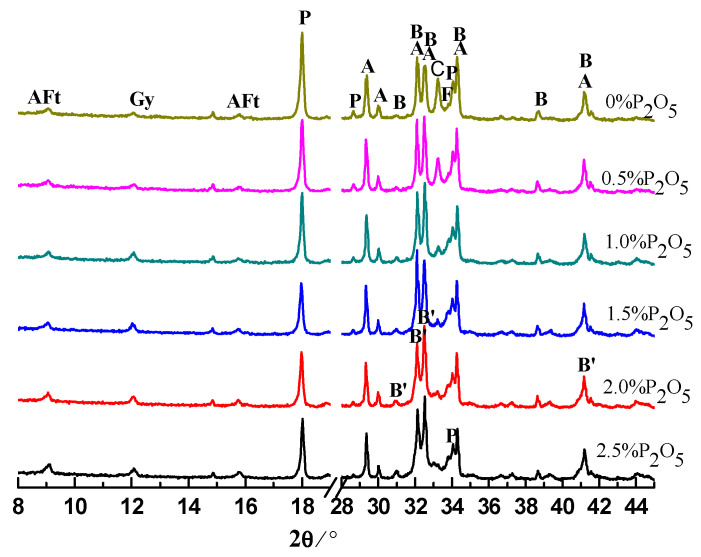
XRD patterns of cement pastes hydrated for 0.5 day. A: C_3_S; B: β-C_2_S; F: C_4_AF; C: C_3_A; P: CH; B′: α′-C_2_S-xC_3_P.

**Figure 4:**
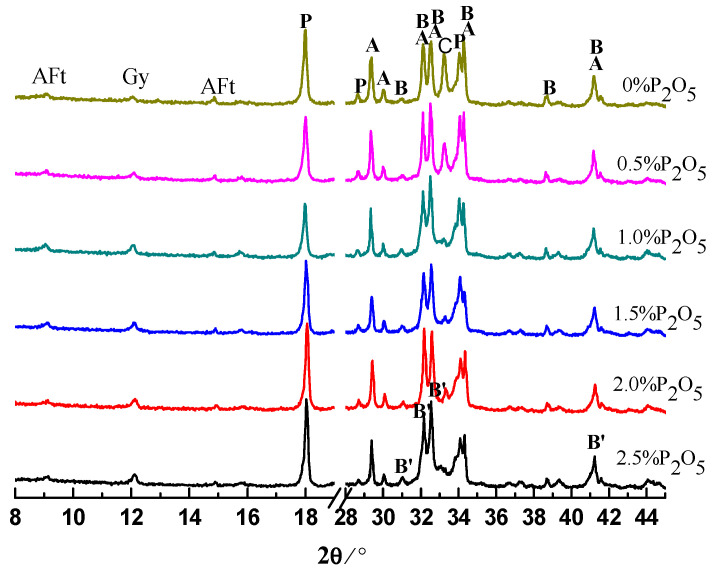
XRD patterns of cement pastes hydrated for 1 day. A: C_3_S; B: β-C_2_S; F: C_4_AF; C: C_3_A; P: CH; B′: α′-C_2_S-xC_3_P.

**Figure 5:**
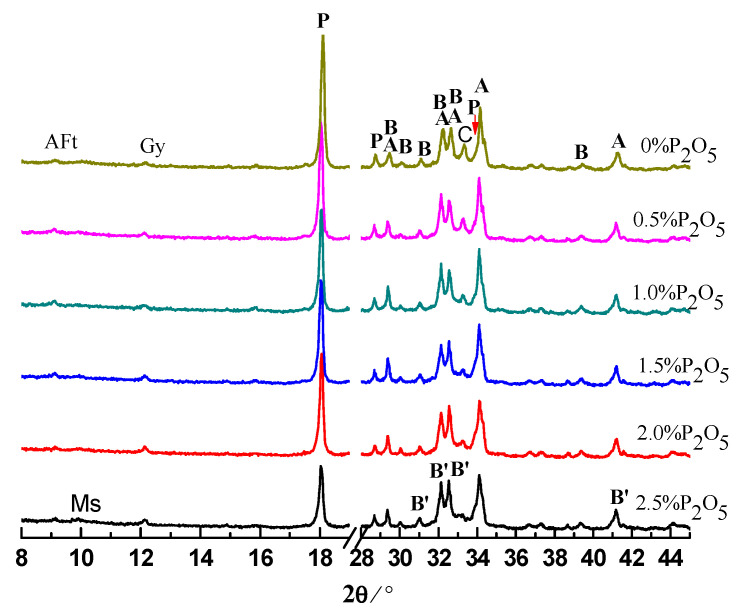
XRD patterns of cement pastes hydrated for 3 days. A: C_3_S; B: β-C_2_S; P: CH; Ms: AFm; B′: α′-C_2_S-0.05C_3_P.

**Figure 6:**
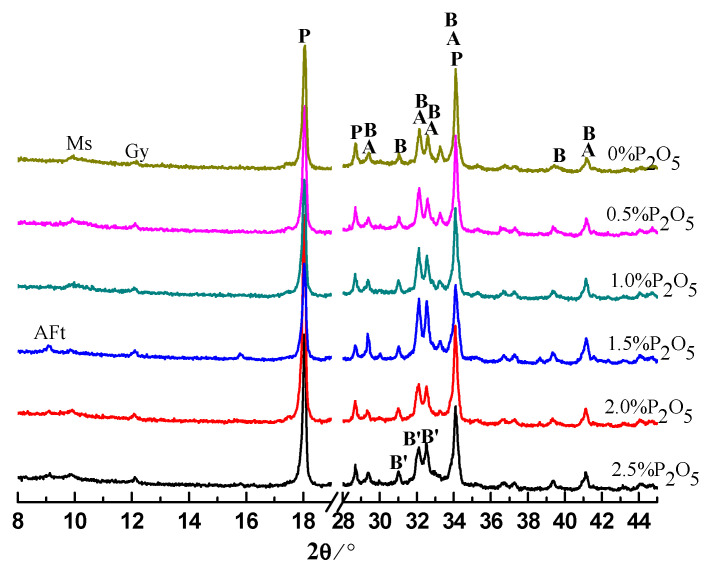
XRD patterns of cement pastes hydrated for 28 days. A: C_3_S; B: β-C_2_S; P: CH; Ms: AFm; B′: α′-C_2_S-0.05C_3_P.

The cement pastes were prepared with water to cement (W/C) ratio of 0.28. The pastes were analyzed by XRD after hydration for 0.5, 1, 3, and 28 days. The results are shown in Figure 3, Figure 4, Figure 5 and Figure 6. Moreover, the typical hydration products (ettringite, calcium hydroxide (CH)) and unhydrated clinker minerals, a new phase of α′-C_2_S-xC_3_P, were detected in the samples containing phosphorous. After hydration of 12 h, the intensity of CH and ettringite (AFt) decreased with the increase of the content of P_2_O_5_. Additionally, the intensity for C_3_S was also gradually weakened with the increase of content of P_2_O_5_.

With the further hydration of cement for 24 h, unhydrated clinker minerals were continuously decreased, corresponding to the enhancement of the intensity of hydration products compared to those samples hydrating for 12 h. However, it is worth noticing that for those samples containing phosphorous, especially for the sample containing 2.5% P_2_O_5_, the intensity of the peak for the α′-C_2_S-xC_3_P was still stronger, which means low reactivity of the cement containing phosphorous. 

Regarding the XRD results for those samples at 3 days, compared to the controlling samples, the intensity for CH significantly decreased due to the existence of phosphorous. This is mainly because phosphorous can inhibit the hydration of cement, which has been supported by the extension of the induction period. Unlike normal conditions, for those samples containing phosphorous, we propose that the new phases of α′-C_2_S-xC_3_P be characterized with low reactivity where intensity of the peak can still be obviously observed.

The products of cement pastes after hydration for 28 days are listed in Figure 6, mainly including CH, Aft, and AFm. It indicates that for the controlling sample, because of the consumption of C_3_S and C_2_S, the corresponding intensity has become far weaker compared to those samples at early stage. However, it is worth noting that the intensity for α′-C_2_S-xC_3_P in those cement pastes containing high phosphorous was still stronger, to some extent suggesting low hydration activity for α′-C_2_S-xC_3_P, which is different from Li’s results that suggested P doping α_H_-C_2_S exerted a high hydraulic reactivity [20]. We speculate that this is mainly because of the distinguished addition of phosphorous weather by chemical agent or phosphorous-bearing materials, causing different effects on the structure of C_2_S, which needs to be further investigated. Moreover, it is interesting that regardless of the length of the curing time, the characteristic peak of calcium phosphate was not detected, which agrees with the previous reports [3,17]. 

### 3.3. Effect of Phosphorous on the DSC-TG of Cement Pastes 

**Figure 7:**
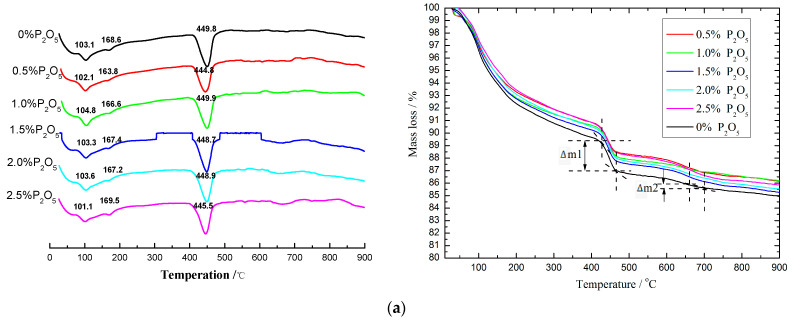

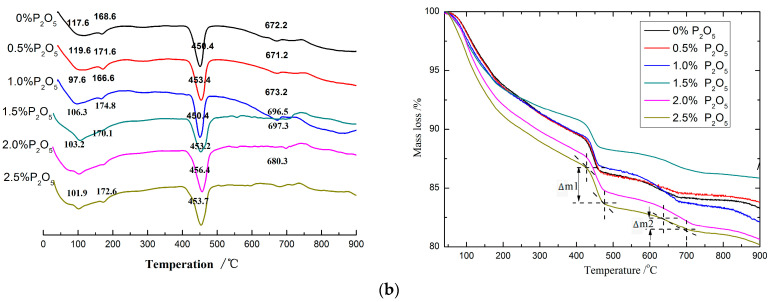
DSC-TG of cement pastes hydrated for (**a**) 3 days and (**b**) 28 days.

Figure 7 shows the DSC results of cement pastes hydrated for 3 and 28 days. The endothermic peaks at 100–105 °C and 165–175 °C were caused by dehydration of C-S-H gel and AFt or AFm. The endothermic peaks at 445–455 °C and 670–700 °C were believed to be brought about by decomposition of CH and CaCO_3_ that was formed by the carbonation of CH [21,22,23], respectively. The results showed that there seems to be no difference in hydration products for cement pastes with and without phosphorus-bearing phases in clinkers.

**Figure 8:**
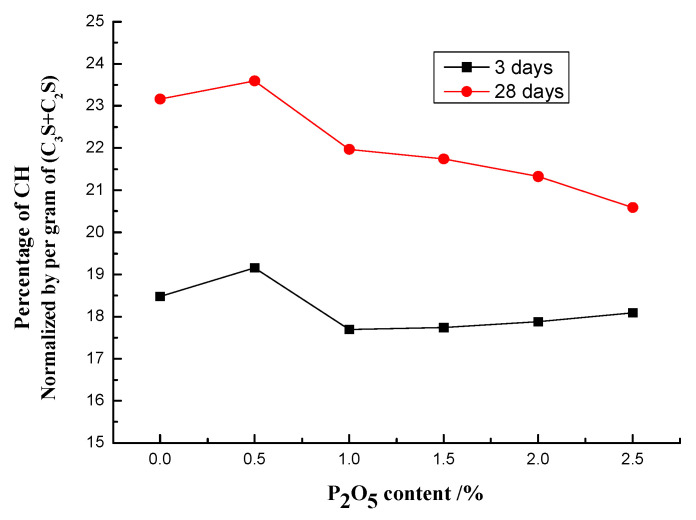
The effect of P_2_O_5_ content in cement clinkers on the formation rate of Ca(OH)_2_ in cement pastes.

Based on the results of TG-DSC, the contents of CH in those samples were calculated by Equation (1). However, accounting for the effective amount of C_3_S and C_2_S whose main hydration products were CH, the normalized value of CH by C_3_S and C_2_S is presented in Figure 8. As mentioned before [24,25,26], because of the mineralization caused by the low dosage of fluorapatite (0.5% P_2_O_5_), compared to the controlling sample, a slight increase can be detected after curing for 3 days. However, when the content of P_2_O_5_ increases to 1.0%, a sudden decrease for CH happens, by 6.5% compared to the plain sample. In addition, when the content of P_2_O_5_ was higher than 1.0%, the CH fluctuated around 18%. Then, after curing for 28 days, except for the 0.5% P_2_O_5_ whose mineralization effect makes CH suffer a slight increase, the continuous increase in the amount of P_2_O_5_ causes a corresponding drop in CH. The results showed that when the content of P_2_O_5_ was 2.5%, the amount of CH at 28 days decreased by 12.1% in contrast to the controlling sample, which matched the development of the compressive strength of cement pastes well, both at 3 and at 28 days.

(1) $$m_{Ca{\left(OH\right)}_{2}}=\left(\frac{\Delta m_{1}}{18}+\frac{\Delta m_{2}}{44}\times \frac{2}{3}\right)\times 74$$

$m_{Ca{\left(OH\right)}_{2}}$: Mass of Ca(OH)_2_;

$\;\Delta m_{1\;}$: Mass change of Ca (OH)_2_;

$\Delta m_{2}$: Mass change of CaCO_3_.

### 3.4. Microstructure of Cement Pastes

**Figure 9:**
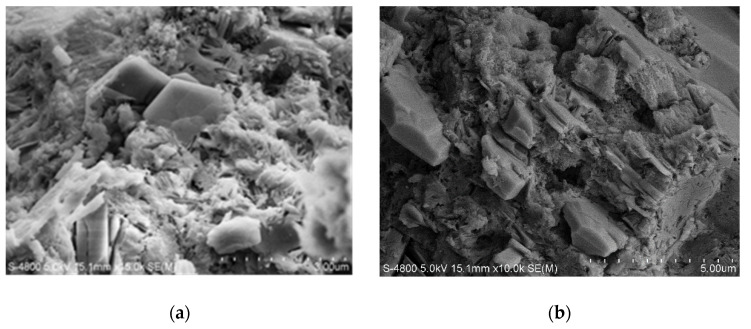

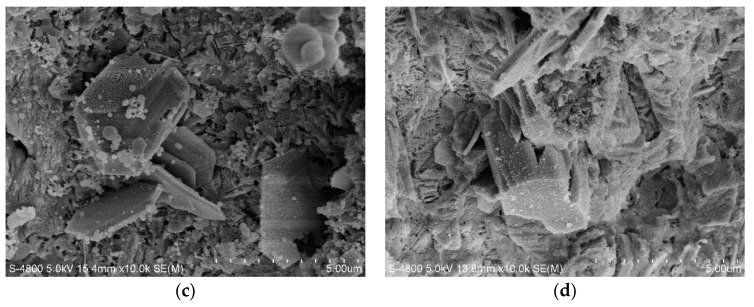
SEM images of cement pastes hydrated for 3 days. (**a**) 0%P_2_O_5_, (**b**) 0.5%P_2_O_5_, (**c**) 2.0%P_2_O_5_ and (**d**) 2.5%P_2_O_5._

Figure 9 shows the effect of content of P_2_O_5_ on the morphology of hydration products at 3 days by SEM. The main hydration products presented mainly included CH and C-S-H. Apparently, with the 0.5% P_2_O_5_, the microstructure of cement pastes was denser compared to the reference one. It is mainly because more C_3_S has been formed due to its mineralization and more hydration products can be obtained, which corresponds to the increase in the strength of cement pastes containing 0.5% P_2_O_5_. For the cement pastes containing both 2.0% and 2.5% P_2_O_5_, its microstructure seems looser compared to the reference sample, which is because the phosphorous will reduce the amount of C_3_S and also lower the hydraulic reactivity of C_2_S, leading to less formation of C-S-H compared to the reference, as the main source of strength. Moreover, it is worth noting that unlike the controlling sample, when the amount of P_2_O_5_ was higher than 2.0%, small particles can be observed on the surface of hydration products, which perhaps introduces new weak interfaces causing a decrease in strength. 

**Figure 10:**
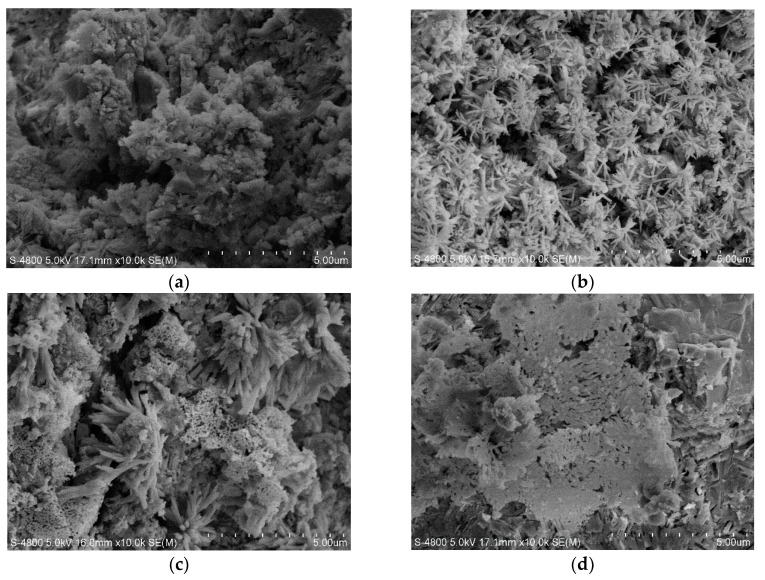
Morphology of cement pastes at 28 days. (**a**) 0%P_2_O_5_, (**b**) 1.5%P_2_O_5_, (**c**) 2.0%P_2_O_5_ and (**d**) 2.5%P_2_O_5_.

**Figure 11:**
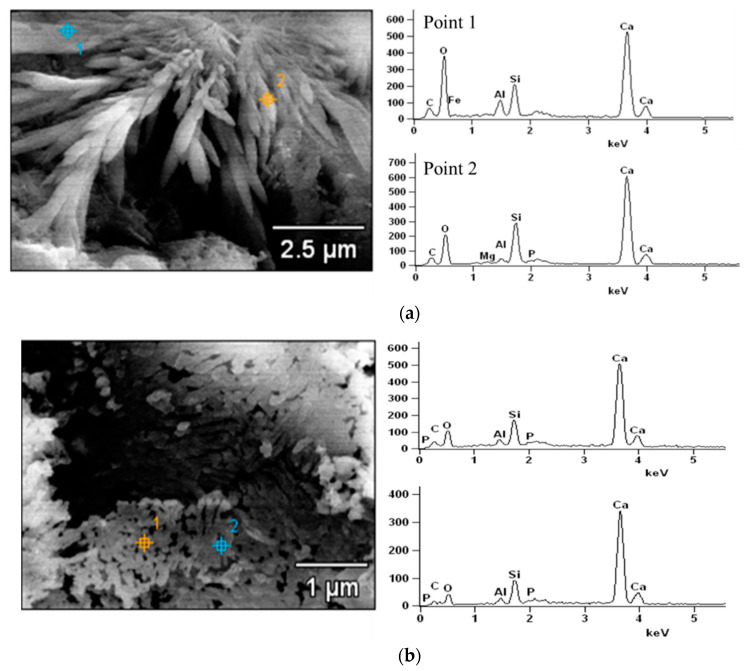
SEM-EDX analysis of hydration products of C-S-H with phosphorous. (**a**) 2.0%P_2_O_5_ and (**b**) 2.5%P_2_O_5_.

Figure 10 presents the morphology of cement pastes with or without P_2_O_5_ at 28 days. It is apparent that the addition of phosphorous will change the morphology of hydration products, especially for the C-S-H gel. Unlike normal cement pastes, those C-S-H gels generated by the phosphorous-containing cement were more fibrous and the microstructure of cement pastes was looser compared to the plain samples. In fact, based on the various previous work on the morphology changes of C-S-H [27], combining with the results, it can be learned that the addition of phosphorous will make C-S-H present as more fibrous with porous small voids in Figure 10d, which matches well with Li’s work. The element composition of C-S-H of cement pastes containing 2% and 2.5% P_2_O_5,_ after curing for 28 days was further analyzed by SEM-EDX (Figure 11). Table 4 shows the chemical compositions of C-S-H in cement pastes with 2.0% and 2.5% P_2_O_5_ by EDX. 

The results showed that the main elements were calcium, silicate, and oxide, which is similar to normal cement pastes. Phosphorous was also detected in C-S-H gel, and the amount correspondingly increased from 0.09% to roughly 0.3%. The amount and polymerization degree of C-S-H in paste containing phosphorous are lower in comparison to those in normal Portland cement [28]. In addition, this is probably the main reason to cause the difference in morphology of C-S-H compared to the reference one [29]. Furthermore, similar to those pastes at 3 days, small particles on the surface of hydration products can be observed at 28 days. In addition, for the 2.5% phosphorous, hydration products will gather. In short, the existence of phosphorous in cement will affect the morphology of hydration products and the microstructure, leading to a decrease in its strength. 

### 3.5. Strength of Cementitious Materials

#### 3.5.1. Compressive Strength of Cement Pastes

**Figure 12:**
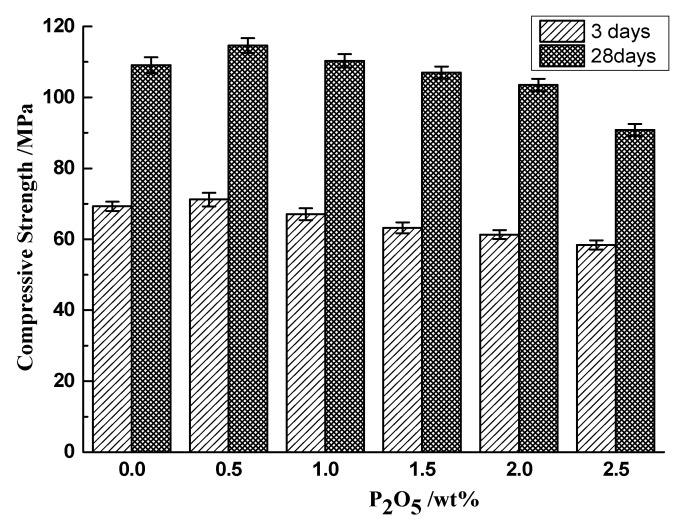
Compressive strengths of the cement pastes prepared with phosphorous-bearing clinkers and hydrated for 3 and 28 days.

The influence of different content of phosphorous on the compressive strength of cement pastes whose W/C was 0.28 at 3 days and 28 days is presented in Figure 12. The results showed that when the content of P_2_O_5_ was 0.5%, the compressive strength of cement pastes at 3 and 28 days was slightly improved by 3.2% and 1.1% compared to the controlling sample. However, with the continuous increase of the P_2_O_5_, the compressive strength of the pastes at 3 days and 28 days was correspondingly decreased. When the P_2_O_5_ went up to 2.5%, the compressive strength of cement pastes at 3 days and 28 days decreased by 15.7% and 16.8%, respectively, compared to the controlling sample. 

#### 3.5.2. Strength of Mortars

**Figure 13:**
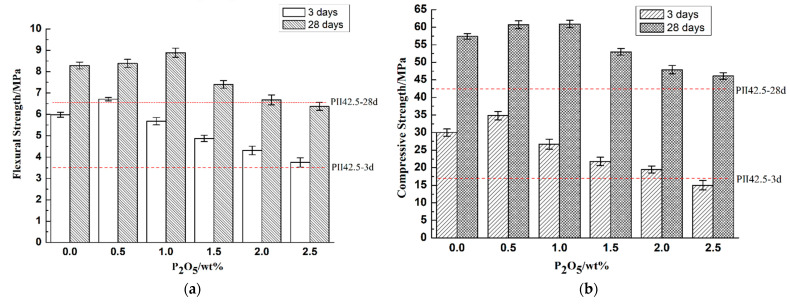
(**a**) Flexural and (**b**) Compressive strength of mortars cured in water for 3 and 28 days.

In addition to the strength of cement pastes, the strength of the mortars containing different contents of phosphorous prepared according to the method of testing cements—Determination of strength GB/T17671-1999 was measured after curing for 3 d and 28 d, as shown in Figure 13.

Regarding with those samples cured for 3 days, the development of both compressive and flexural strength of mortar were basically consistent with the compressive strength of cement pastes; specifically, it will be slightly promoted with 0.5% phosphorous and then gradually decreased with the increase of phosphorous. However, for those samples cured for 28 days, except for 0.5% P_2_O_5_, the flexural and compressive strength of mortar containing 1.0% P_2_O_5_ increased by 7.2% and 6.1% respectively. Then, the strength of mortars exerted a significant decrease with the increase of phosphorous. When the content of P_2_O_5_ was 2.5%, the flexural and compressive strength were separately reduced by 23.2% and 19.7%. However, it is worth noticing that according to the requirement of Chinese standards for the cement industry, even the P_2_O_5_ was high at 2.0%; it can still meet its requirement of P·II 42.5, which means that it is feasible to use phosphorous limestone as raw materials to produce Portland cement. 

### 3.6. Discussion

It is well known that cement hydration is a dissolution-participation process, which is closely related to the properties of cement clinker, particularly for mineral composition and its crystal structures. The existence of phosphorous in cement surely exerts significant influence on the hydration process, representing changes in strength. At the early stage of 3 days, the strength of cement pastes and mortars containing 0.5% P_2_O_5_ can be enhanced due to its mineralization effect, causing more C_3_S formation, meaning more C-S-H gel can be obtained. However, at later stage of 28 days, the strength of mortars containing 1.0% P_2_O_5_ were also improved. It can be speculated that despite a decrease in C_3_S, more C_2_S can be formed, which is more positive for promotion at a later stage. However, with an increase of P_2_O_5_, on the one hand, the amount of C_3_S will decrease and more β-C_2_S will transfer into α′-C_2_S. Furthermore, phosphorous will dissolve into minerals and participate in the hydration. As a result, with the hydration of silicate phase, more phosphorous in solid solution will come out, which may react with Ca^2+^ to form Ca_3_(PO_4_)_2_ precipitated on the surface of hydration products and anhydrate clinker particles, inhibiting its hydration and prolonging the induction period as showed in the hydration heat flow. However, according to the XRD results, no Ca_3_(PO_4_)_2_ has been detected, which we speculate is for two reasons: (1) not enough Ca_3_(PO_4_)_2_ has been used to be detected; (2) as mentioned, the existence of phosphorous will change the structure of silicate phases, especially for C_2_S from β-C_2_S gradually to α′-C_2_S, leading to the different morphology of C-S-H gel, as shown in Figure 10. In fact, the hydration heat flow ratio at the accelerated period to some extent indicates that the growth ratio of C-S-H slightly decreased, which was constant with Wu’s reports that phosphorous can affect the crystal structure of C_2_S and reduce its reactivity [30]. Furthermore, unlike normal cement hydration processes, the aforementioned results explain that the use of phosphorous-bearing limestone exert a greater impact on the property of C_2_S than on C_3_S. The formation of a new phase of α′-C_2_S-xC_3_P whose hydration activity will dramatically decrease is demonstrated based on the results of XRD. This is opposite to previous results regarding the influence of the addition of phosphorous-related chemical analytic agents on the C_2_S structure and its hydraulic hydration. However, accounting for instances [27] where different ions and concentrations will change the morphology of C-S-H, specifically the addition of phosphorous in the solutions will make the C-S-H have more fibrils and also be more porous with small voids, which agreed well with other researchers’ work [20,31]. Therefore, it is apparent that the influence of phosphorous introduced separately by industrial raw materials and chemical analytical agents will exert different effects on both the structure and activity of C_2_S, but has similar morphology of C-S-H. In short, the large amount of phosphorous in those mineral phases, particularly for C_2_S by α′-C_2_S-xC_3_P, will undoubtedly dissolve into solutions, prolong the induction period, decrease the hydration heat flow, and change the microstructure of cement pastes, leading to the decrease in its strength. Again, unlike the phosphorous-bearing cement clinker introduced by chemical agents, it will be more effective to study the hydration and strength development of cementitious materials prepared with phosphorous-bearing clinkers.

## 4. Conclusions

A systematic investigation about the mechanical properties of phosphorous-containing cement and its hydration mechanism was carried out. Specifically,

(1) When the P_2_O_5_ in the clinker is 0.5%, both compressive and flexural strength of cement pastes will be improved separately at 3 and 28 days, while with the continuous increase of P_2_O_5_, the strength of cement pastes will decrease; for mortars, when the content of P_2_O_5_ is 1.0%, the compressive and flexural strength of mortars for 28 days can still be promoted. However, for others, the addition of phosphorous will be harmful for the strength of mortars. Moreover, even the P_2_O_5_ in the clinker is as high as 2.0%, and the cement can still meet the requirements of P·II 42.5 (Chinese standard).
(2) The existence of phosphorous in the clinker will seriously prolong the induction period and decrease the total hydration heat. Despite those small particles on the surface hydration products, no new hydration products for phosphorous-containing cement have been detected. However, C-S-H gel for the pastes containing phosphorous will present as fiber-like, and it is also thicker than the plain one. Moreover, the microstructure for cement pastes containing phosphorous will be looser compared to normal cement pastes.
(3) However, unlike C_2_S, it can be speculated that the hydration reactivity of the new phase of α′-C_2_S-xC_3_P is rather lower based on the XRD results, leading to the less formation of hydration products and poor structure of cement pastes or mortars, directly causing poor mechanical property.
(4) A special type of P·O 42.5 cement has been produced, which can prove the workability of the use of low-grade phosphorous limestone as the raw materials for cement production, not only providing a significant guidance for actual industrial cement production but also greatly cutting the production cost. However, its application may only be aimed at projects that require a long setting time.

## Figures and Tables

**Table 1:** Influence of P_2_O_5_ on the content of C_3_S and C_2_S in clinkers /wt.%.

P_2_O_5_	0	0.5	1.0	1.5	2.0	2.5
C_3_S	53.06	53.64	47.22	45.37	44.83	42.69
C_2_S	23.52	21.04	31.55	32.54	31.40	30.78

**Table 2:** The proportion of cement paste and mortars.

	Cement/g	Sand/g	Water/g
Cement pastes	450	0	126
Mortars	450	1350	225

**Table 3:** Eigenvalues of hydration heat flow released by phosphorus-bearing cements.

P_2_O_5_ in Clinker/%	Total Hydration Heat at 3 days/J·g^−1^	Time at End of Induction/h	Minimal Hydration Heat Flow/mW·g^−1^	Time at End of Acceleration Period/h	Maximum Hydration Heat Flow /mW·g^−1^
0.0	151.7	2.17	0.67	7.38	2.06
0.5	157.3	2.68	0.49	8.75	1.88
1.0	158.0	3.03	0.48	9.38	1.71
1.5	158.3	3.32	0.45	9.38	1.72
2.0	141.9	3.72	0.42	10.96	1.52
2.5	142.7	5.45	0.40	12.73	1.55

**Table 4:** Chemical composition of C-S-H in cement pastes with 2.0% and 2.5% P_2_O_5_ by EDX/wt.%.

P_2_O_5_ in Clinker /%		O-K	Al-K	Si-K	P-K	Ca-K
2.0	Point 1	66.61	3.00	5.53	-	22.37
	Point 2	59.94	0.93	8.35	0.09	30.34
2.5	Point 1	51.08	1.64	7.55	0.26	39.46
	Point 2	43.26	1.65	8.01	0.35	46.72

## Data Availability

The data presented in this study are available on request from the corresponding author.
